# Supplementary material for: Are carbon emissions trading and green financial instruments synergistic? -Comprehensive quantitative research based on content analysis
Source: PLoS One. 2024 Mar 7;19(3):e0298601. doi: 10.1371/journal.pone.0298601 (PMC10919601; doi:10.1371/journal.pone.0298601)
Supplement: S1 Data — (ZIP) [file pone.0298601.s001.zip › Data/地级市Z_F工作报告.zip!/╝¬┴╓/░╫╔╜╩╨/└┤╫╘╣1⁄2╓┌║┼í╛┬φ┐╦╩2╛▌═°í┐_2018.docx]

各位代表：

　　现在，我代表市政府，向大会作政府工作报告，请予审议，并请市政协各位委员提出意见。

　　一、2017年工作回顾

　　刚刚过去的一年，是实施“十三五”规划承前启后的重要之年和新一届政府的开局之年，是极不平凡的一年，各项改革发展任务极其艰巨繁重。我们把喜迎党的十九大、学习党的十九大、贯彻党的十九大作为主线，接受了中央巡视“回头看”、中央环保督察、国务院安全生产督察、省扶贫领域专项巡视等政治检验，在省委、省政府和市委坚强领导下，在市人大、市政协监督支持下，团结带领全市广大干部群众克难攻坚，办成和解决了许多大事难事，实现了许多重要领域和重点工作的突破，取得了绿色转型全面振兴的阶段性成果。

　　（一）全力以赴稳增长，经济发展稳中向好。保持定力、精准发力，经济运行稳中有进、稳中向好。预计实现GDP730亿元，增长5.5%；规上工业增加值378.5亿元，增长0.5%；固定资产投资682亿元，增长3%；服务业增加值272亿元，增长12%；社会消费品零售总额316亿元，增长8%；城乡居民人均可支配收入分别达到22550元和10360元，均增长6%。工业稳步发展。深入开展重点企业“一对一”精准帮扶专项行动，建立市级领导包保亿元以上投产达效项目和“周调度、月分析”机制，帮扶企业稳产促销、做大做强，新增1个中国驰名商标和9个省名牌，规上企业利润18亿元。投资187亿元实施工业技改项目420个，开复工施慧达药业新厂、佳泰200万只铝车轮等135个重点工业项目，春柏药业异地改建等37个项目竣工投产。农业提质增效。全力推进率先实现农业现代化，启动“一区三园”建设，做好“一增一减”，新增特色经济作物24.4万亩，调减籽粒玉米30.1万亩，高效作物占有率达60%以上。成功举办首届长白山道地药材交易会，抚松成功创建国家有机食品生产基地建设示范县，万良人参市场升级为国家级专业市场，江源蓝莓国家农业标准化示范基地获批。江源、长白被评为省级电商进农村示范县，在全省率先实现示范县全覆盖。新增国家星级休闲农业企业6户、省级农业产业化龙头企业7户。实现特产业产值140亿元，增长10%；农产品加工销售收入276亿元，增长9%。现代服务业加快发展。集中力量打好服务业发展攻坚战，推进鹏翔物流二期等46个重点项目，新增限上商业和规上服务业企业19户。圆满完成宽带中国示范市创建任务，实现行政村光纤宽带全覆盖。成功举办“长白山之夏”、“长白山之冬”旅游节，各类微博话题阅读量和网页点击量达3亿余次。东北首个“摄影小镇”落户长白，临江鸭绿江国家级水利风景区获批，抚松、靖宇、临江、长白分别被评为全国十佳宜居县、人文生态旅游基地、森林旅游示范县、最美休闲自驾游目的地。白山知名度和美誉度大幅提升，接待国内外游客突破千万人次，实现旅游社会总收入150亿元，分别增长15%和25%。财政收入质量稳步提升。实现地方级财政收入27亿元，其中国税、地税分别组织收入10.2亿元、11.4亿元，按可比口径分别增长13.1%和16.3%，税收占地方级财政收入比重74.8%，同比提高7.2个百分点。

　　（二）多措并举调结构促转型，新旧动能转换不断加快。坚持扩大优质增量与调整优化存量并举，经济结构进一步优化，三次产业结构由8.2：55.7：36.1调整为8.5：54.2：37.3，“新五样”占GDP和财政收入比重分别达到64.4%和48.6%。白山成为全国首家绿色转型示范区。项目建设成果丰硕。大力实施“冬季三项促投资活动”和项目“三早”行动，开展项目大巡检，超常举措破解土地、拆迁、资金等瓶颈制约，实施总投资112亿元的漫江生态文化旅游综合开发、总投资100亿元的长白山广泽旅游度假小镇等5000万元以上项目232个，其中亿元以上项目154个，增长32.6%和24.2%。项目建设呈现数量多、大项目多、产业项目多、民营资本和外来投资比重大等特点，我市在全省投资和项目建设工作会议上介绍做法。科技创新步伐加快。推进“院士专家白山行”、“百所高校进白山”活动，成立长白山人参等3个研究院、抚松华润和善堂等9个院士工作站。我市被授予“中国硅藻土之都”称号，省硅藻土产品质量监督检验中心和唯一的国家级饮用水质量监督检验中心落户白山，硅藻土行业及两类制品标准在我市发布。新增3户国家高新技术企业和7户省首批科技小巨人企业，34个项目列入省级科技发展计划。民营经济发展势头强劲。坚持抓生成、抓培育、抓长效、抓服务，开展小微企业帮扶、市场主体培育、“小升规”专项行动和“万名创业者、万名小老板”培训，搭建双创孵化基地，成立白山商会。助保金池发放贷款4140万元，股权出质、商标权质押和动产抵押三大平台助企融资19.5亿元。全市新增市场主体1.5万户，增长14.5%，民营经济主营业务收入突破2000亿元大关，对GDP和财政收入贡献率分别达到52.5%和73.6%。我市被确定为首批东北地区民营经济发展改革示范市。

　　（三）全面深化改革开放，发展动力和活力加速释放。坚持全方位、多层次、宽领域，进一步深化改革扩大开放。供给侧结构性改革加快推进。关闭退出4处煤矿，去产能57万吨；房地产去库存10万平方米，货币化安置率和去库存率分别达到100%和10%；积极推进类金融集聚区和农村产权交易中心建设，组建市融资平台公司，引进50亿元城市发展基金和百亿元健康企业股权基金，皇封参成功在新三板上市。重点领域改革取得新突破。机关事业单位养老保险、文化市场综合执法和公车改革完成，国有林场改革方案获批，城市管理综合执法和投融资体制改革加快推进，城市公立医院综合改革全面启动。农村土地“三权分置”完成确权登记。国企“三供一业”分离移交全部签署协议。对外开放持续深化。启动鸭绿江开发开放经济带建设，高标准编制总体规划和沿鸭绿江旅游公路实施方案，建成长白10公里景观带，开工临江国际演艺广场、神龟湾景区和浑江满族风情园。白山经开区新区获省政府批复。建立与浙江湖州对口合作机制。抚松成功创建国家级出口木制品质量安全示范区。成功举办2017中国?白山“生态文明?绿色转型”论坛。启动建设白山海关。招商引资成效显著。大力开展“冬春会战”，突出“六个围绕”，市县两级党政领导多次率队赴京浙沪等地开展精准对接和集中招商。积极参加东博会、吉商大会，成功举办硅藻土新材料、医药健康产业论坛，乌镇世界互联网大会白山国医馆上线运营。组建市经合局，选派百名后备干部到发达地区挂职招商，完善“五个一”、联席会议、考核评比等机制，形成招商引资强大合力。成功引进总投资150亿元的复华长白山国际度假区、总投资120亿元的天人合一“长白天地”等157个项目。引进域外资金、实际利用外资分别增长9%、10%。

　　（四）加大基础设施建设力度，城乡面貌发生显著变化。坚持规划、设计、施工、征拆、融资同步推进，过去一年是我市出台城市规划、实施城建项目、征地拆迁规模、投融资力度最多最大的一年。中心城区品位提升。实施城市三年综合整治计划，成立市城乡规划委员会，大力推进“五化”工程，投资15亿元实施净畅绿亮美项目64个，购置环卫机械化设备1179台，开展城市净化攻坚月、道路交通秩序整治大会战、城市东出口和北线环境整治等活动，实施“日晨检、周通报、月总结”，清理垃圾35万吨，红旗街等14条标准化示范街路面貌一新，碱厂沟河、月牙河等4条黑臭水体治理步伐加快，建设浑江干流堤防6公里；东台路、喜丰路等6条断头路全线贯通，南北线22处互通道路实现硬覆盖；城市出入口、滨江景观带、城市慢行系统、主街路亮化提升工程全面开工；366栋老旧和弃管小区楼房“保基本”改造和临街楼体“穿衣戴帽”工程有序推进，3505户21万平方米棚改任务超额完成。推进“多规合一”，高标准完成城市总体规划和13个专项规划，形成完整规划体系。加大城市管理力度，制定物业、市容环境卫生等4个条例，拆除违建24.3万平方米。强力推进征地拆迁，一大批多年想解决而未解决的“钉子户”、“老大难”问题有效化解，征收3562户2817亩。县城建设提档升级。实施抚松道路改造、靖宇棚改、长白文化体育综合体、临江全民健身中心等一批重点城建项目，松江河镇被列为国家级特色小镇。农村环境持续优化。启动农村人居环境改善四年行动计划，构建全域旅游节点村落，高标准创建22个省级重点村、10个省级美丽乡村示范村，打造5100户美丽庭院、10600户干净人家，完成改厕5500户。大交通建设全面提速。辉白高速白山段完成主体工程，白临高速完成工可研前置要件，松长高速完成工可研编制。沈白高铁、白山机场前期工作进展顺利。白山至三道沟公路全线贯通，错草至三道沟、靖宇至江源和沿鸭绿江旅游公路加快推进，新改建农村公路212公里。

　　（五）大力推进生态文明建设，环保专项治理取得实效。全面落实环境保护“党政同责、一岗双责”，深入贯彻中央环保督察各项工作部署要求，实施清洁空气、水体、土壤行动，集中解决原麝保护区等165件群众反映强烈环境问题，淘汰燃煤小锅炉39台、黄标车2745台，完成率居全省前列。坚持“四个不放过”和“四个第一时间”要求，全部办结中央环保督察组交办案件。认真做好“绿盾2017”专项行动迎检工作，整改保护区问题311个。全面推行“河长制”，228条河流设立市县乡村四级河长。建立大气、水污染防治联席会议和重污染天气预警研判等机制，较好完成了履行自然资源资产管理和生态环境保护责任审计工作。新增抚松国家地质公园、龙山湖国家级森林公园和老梁山、五间房2个省级森林公园。市区空气优良天数达到300天以上，重点流域水质保持3类以上标准，集中式饮用水源地水质全部达标，水环境质量居全省前列。全市森林覆盖率达到84.1%。抚松、临江、长白入选全国百佳深呼吸小城，长白荣登榜首。

　　（六）坚持保障和改善民生，人民生活水平不断提高。始终坚持新增财力80%以上用于民生。实施脱贫攻坚“五个一批”工程，投入17.7亿元推进214个产业扶贫项目，解决1.9万户农村居民供水安全问题，改造1526户贫困户危房。全面开展与吉林市扶贫协作，脱贫7879人，退出贫困村22个，贫困发生率下降2.4个百分点。37件民生实事完成34件，因规划调整等原因有3件未全部完成，根据群众诉求和期盼新增149栋弃管小区老旧居民楼改造等5件实事。投入3亿元推动五大保险扩面提标，基金监管有序开展，社保“一卡通”信息采集90万人，覆盖率达到75%。城镇新增就业4.4万人，农村劳动力转移就业10.7万人，城镇登记失业率控制在4%以内。累计救助城乡困难群体15.8万人次，发放救助金和低保金4亿元，城乡低保标准分别提高6.3%和23.6%。“健康快车光明行”免费为1050名白内障患者进行复明手术。成功举办长白山文化、抗联文化高端论坛和“四保临江”战役胜利70周年纪念大会，《陈云与四保临江》纪录片在央视黄金时段播出，《民族记忆》和《鸭绿江纪事》在我市完成拍摄，《白山史话》栏目荣获全国市县电视台推优评比一等奖。完成文化惠民演出400场、放映农村公益电影6000场。启动建设文化体育艺术中心，长白山职业技术学院新校区和怡康养老综合体主体完工，短道速滑馆投入使用。圆满举办市八运会，成功申办省第八届少数民族运动会。推进“安全生产责任深化年”和“五个一工程”，排查整改隐患2万余项，实现连续21个月无重大事故，得到国务院安委会督察组充分肯定。实施食品药品安全放心工程，未发生重大食安事件。连续38年无重大森林火灾。立足“事要解决、案结事了”，化解维多利亚花园烂尾工程等一批重大信访积案，信访总量大幅下降，实现重大会事期间进京非访零登记。成功破获建国以来我省涉案金额最大的“9.22”特大跨国电信网络诈骗案，圆满完成十九大安保维稳任务。开展“打黑除恶”、“打盗骗保平安”、“雷霆扫毒”等专项行动，刑事案件发案率下降21.7%。公共安全视频监控联网应用平台暨“雪亮工程”开工建设，成为全省首个获国家重点支持的城市。开展双拥“六进”活动，推动军民融合深度发展。

　　（七）加强民主法制和政府自身建设，工作效能和水平进一步提升。全面启动法治白山建设，出台《白山市人民政府重大行政决策程序规定》、《加快法治政府建设实施细则》、《依法行政制度体系建设工作方案》，提请市人大常委会审议地方性法规草案1部。深入推进“七五”普法，完成全国重大执法决定法制审核试点创建工作。办理人大代表建议和政协委员提案134件，办结率100%。推进“两学一做”学习教育常态化制度化，持之以恒落实中央八项规定精神和省委、市委有关规定，驰而不息抓作风、纠“四风”，保持机构编制和“三公”经费零增长，楼堂馆所零开工。严格落实“两个责任”，履行“一岗双责”，深入开展政府系统党风廉洁建设和反腐败斗争，强化行政监察和审计监督，努力从源头上预防和治理腐败。加快“放管服”改革，推进“四单一网”和“多证合一”，实现“双随机一公开”全覆盖，政务服务“一张网”上线运行，支持抚松扩权强县改革下放134项行政审批权。深入实施“软环境建设年”，用硬措施整治软环境。建立投资项目快速审批机制，落实“首办负责、并联审批、全程代办、限时办结”，确保工业项目在35个工作日内、房地产等基础设施类项目在55个工作日内完成所有审批事项，企业办照和审批时间压缩85%和35%以上。

　　各位代表！面对复杂多变的外部环境和艰巨繁重的改革发展任务，我市经济社会发展取得的新成就来之不易。这是省委、省政府高度重视、亲切关怀的结果，是市委坚强领导、科学决策的结果，是市人大、市政协和社会各界监督支持、倾力帮助的结果，更是全市人民迎难而上、奋力拼搏的结果。在此，我代表市政府，向全市各族人民，向驻白山人民解放军指战员、武警部队官兵、公安司法民警、中省直单位，向各民主党派、工商联、人民团体、无党派人士、离退休老同志，向关心支持白山发展的各界朋友，致以崇高的敬意和衷心的感谢！

　　在肯定成绩的同时，我们也清醒地看到存在的问题。一是经济下行压力较大，稳中向好发展的基础还不牢固，质量效益还不够高，个别指标增速没有达到预期。二是转方式、调结构、促转型任务依然繁重，新旧动能转换仍需持续发力，“老三样”向“新五样”转型仍需提档加速。三是交通、市政等重要基础设施仍存在短板，城市品位、集聚辐射功能和承载力与加快发展、改善民生仍不适应。四是发展理念和体制机制还不能较好适应转型升级需要，改革力度和深度仍需拓展，创新支撑发展能力仍需加强，对外开放领域和层次仍需提升。五是民生保障压力较大，脱贫攻坚任务艰巨，生态环境修复治理任重道远，安全生产和边境安全等形势严峻。六是少数政府工作人员发展意识、大局意识、服务意识、担当意识还不够强，学习能力和执政本领不足，庸、懒、散以及不作为、乱作为，不敢担当、不善担当等现象还不同程度存在。对这些问题，我们一定高度重视，采取有效措施，切实加以解决。

　　二、2018年工作安排

　　2018年是全面贯彻落实党的十九大精神的开局之年，也是决胜全面建成小康社会、加快绿色转型全面振兴的关键之年。政府工作总体要求是：高举习近平新时代中国特色社会主义思想伟大旗帜，全面贯彻落实党的十九大精神，牢牢把握“五位一体”总体布局、“四个全面”战略布局，深入贯彻新发展理念，全面落实省委“三个五”战略和中东西“三大板块”战略部署，坚持生态立市、产业强市、特色兴市，以满足人民日益增长的美好生活需要为目标，以建设现代化经济体系为指引，以推进供给侧结构性改革为主线，坚持稳中求进总基调，深入实施“3331”发展战略和“十大工程（战役）”，统筹推进稳增长、促改革、调结构、惠民生、防风险各项工作，不断开创绿色转型全面振兴新局面。

　　综合考虑各种因素，全市经济社会发展主要预期目标是：GDP增长6%，规上工业增加值增长2%左右，服务业增加值增长12%，地方级财政收入增长2%，社会消费品零售总额增长8%，城乡居民人均可支配收入与经济增长同步，城镇登记失业率控制在4%以内，单位GDP能耗下降3.2%，碳排放强度下降3.96%。

　　围绕实现上述目标，重点抓好以下工作。

　　（一）贯彻新发展理念，加速建设现代化经济体系。坚持以提高发展质量和效益为中心，加快构建特色鲜明的现代产业体系，不断推动质量变革、效率变革和动力变革。

　　持续深化供给侧结构性改革。把发展经济的着力点放在实体经济上，把提高供给体系质量作为主攻方向，突出改造升级“老三样”，突出发展矿产新材料产业，突出打造大生态、大健康、大旅游、大服务、大文化融合发展的绿色产业体系。抓好“三去一降一补”，全部完成煤炭去产能任务，力争房地产去库存率达到15%，大力发展股权和债券融资，推动施慧达等6户企业主板上市，持续实施降本增效行动，稳步推进国企“三供一业”分离移交。实施民营经济提升工程，围绕创建东北地区民营经济发展改革示范市，研究促进民营经济发展新举措，持续开展市县两级领导帮扶包保重点民企行动，完善联系企业制度，构建“亲”“清”政商关系，力争新增市场主体7000户，增长7.8%，民营经济主营业务收入增长7%。实施“白山工匠”培育工程，围绕转型升级建设知识型、技能型、创新型劳动者队伍，弘扬劳模精神和工匠精神，营造劳动光荣的社会风尚和精益求精的敬业风气。

　　着力实施产业项目建设年。牢固树立“今天的项目就是明天的产业、今天的招商就是明天的项目”理念，大力开展“冬季三项促投资活动”，实施5000万元以上项目167个，重点推进葵花药业医药健康产业园等85个重大产业项目，竣工农夫山泉200万吨天然水等40个项目，滚动谋划亿元以上项目135个。持续打好招商引资“冬春会战”，抓好产业链招商、新业态招商、总部经济招商、重大平台招商，力争在引进50亿、超百亿的龙头型、旗舰型、基地型项目上取得新突破。加快白山经开区新区建设，完成起步区1平方公里征地，开工“一横一纵一辅”道路，建成双创孵化基地，依托新区打造类金融和总部经济集聚区。深化与浙江湖州对口合作，建设浙江产业园，有效承接产业转移。建立招商引资大数据库，推进信息化招商。加强招商人员培训，充分发挥白山商会和挂职招商干部作用。引进域外资金增长5%。

　　着力实施工业经济提振行动计划。坚持扩量与提质并举，启动百亿技改、百企提升“双百”工程，稳定工业经济增长，提高整体实力和竞争力。实施分类指导，实现帮扶工作常态化、精准化、责任化，力争70%以上“双停”企业复产复工，新增规上企业20户。加快推进120个重点工业项目，力争修正200万吨山泉水、金豹木业10万套硅藻土复合门等30个项目竣工投产。鼓励企业争创名牌名标，提升品牌竞争力，新增省级以上名牌4个。

　　打好服务业发展攻坚战。坚持生产性与生活性服务业并重、现代与传统服务业并举，推进怡康养老综合体等45个重点项目，抓好白山综合物流枢纽中心、靖宇和松江河物流园区建设，打造2个特色智慧商圈。实施“旅游+”战略和旅游产品提升工程，持续打造“长白山之夏”和“长白山之冬”旅游品牌，推进漫江生态文化旅游综合开发等重点项目，围绕红色旅游、冰雪旅游、乡村旅游等打造8条精品旅游线路，串珠成链、串线成面。开工建设中朝国际旅游风情带沿鸭绿江旅游公路30公里示范路。全面推广“1+3+N”旅游管理体制，争创国家全域旅游示范区。接待国内外游客人数、旅游社会总收入分别增长13%和20%。

　　（二）大力实施乡村振兴战略，加快率先实现农业现代化。按照产业兴旺、生态宜居、乡风文明、治理有效、生活富裕的总要求，建立健全城乡融合发展体制机制和政策体系。

　　大力发展生态效益型特色农业。扎实推进农业供给侧结构性改革，持续做好“一增一减”，新增特色经济作物6万亩，调减籽粒玉米8万亩，高质量打造北佳中药材、金田蓝莓等30个特色农产品示范基地。积极培育新型农业经营主体，新增6户国家星级休闲农业企业。实施农业品牌推进年工程，做大做强人参、蓝莓、道地中药材等品牌。实现特产业产值155亿元，农产品加工销售收入突破300亿元，均增长10%。

　　深化农业农村改革。推进农村金融体制改革，加快建设“三支柱一平台”。深化农村土地和集体产权制度改革，完善“三权分置”制度，保障农民财产权益，实现农村土地流转中心和产权交易市场全覆盖。促进农村一二三产业融合发展，支持和鼓励农民就业创业，拓宽增收渠道。培养造就一支懂农业、爱农村、爱农民的“三农”工作队伍。

　　抓好美丽乡村建设。深入推进农村人居环境改善行动计划，实施“双十双百双千”工程，建成20个省级重点村民生项目，建设100个生态宜居示范村、100个整洁宜居美丽乡村，创建2000个美丽庭院和干净人家星级文明户。扎实推进厕所革命，完成改厕6000户，农村垃圾处理率达到80%。注重保护好自然生态、田园风光，守住历史风貌、乡土气息，打造一村一景、一村一韵、各美其美的乡村景致。

　　（三）深入推进改革创新开放，不断激发全社会创造力和发展活力。蹄疾步稳推进全面深化改革，发展更高层次的创新型和开放型经济，进一步释放发展动力和活力。

　　持续深化重点领域和关键环节改革。大力推进“放管服”改革升级版，成立投资项目审批服务代办中心和中介服务超市，探索实施“最多跑一次”和“零跑动”改革，力争覆盖20%以上行政审批事项。加快建设政务服务“一张网”，推进政务信息系统整合共享，适时启动智慧城市建设，让数据多跑路，企业和群众少跑腿。深化财税体制改革，推进财政事权和支出责任划分改革，建立全面规范透明、标准科学、约束有力的预算制度，有效防控债务风险。统筹推进投融资体制、国有林场、城市公立医院、供销合作社等各项改革。

　　深入实施创新驱动发展战略。在更大范围、更广领域、更深层次推动创新，精心打造经济社会发展核心引擎。实施“小升高、小壮大、小做强”工程，新增创新型科技企业10户。进一步深化与清华、吉大等高校院所合作，实施30个创新项目，推动10个产学研合作项目落地。深化科技体制改革，围绕高水平研发平台建设、高层次团队引进、高科技项目争取等方面，制定出台建设创新型城市相关政策，建立以企业为主体、市场为导向、产学研深度融合的技术创新体系。坚持培养和引进并重，更加注重柔性引进、项目引进、专项资助引进，实施高层次人才引进计划，持续开展“院士专家白山行”和“百所高校进白山”活动，聘请国内外顶级专家担任科技、经济顾问，力争在引进院士和“国家千人计划”上有新突破。

　　全方位扩大对外开放。加快建设鸭绿江开发开放经济带，全力构建绿色转型振兴平台、改革开放创新平台、兴边富民固防平台，打造我省向东向南开放腹地、东部绿色产品集散地、连接东北东部经济带重要节点城市。积极推进长白国家重点开发开放试验区，长白、临江边境经济合作区和互市贸易区建设。加快建设白山海关，推动省级矿泉饮品外贸转型升级示范基地获批。

　　（四）突出抓重点、补短板、强弱项，加快城市建设步伐。坚持完善功能、提升品位，统筹推进城乡建设，打造宜居宜业宜游城市，加快实现城市环境质的提升。

　　抓好中心城市建设。坚持有所先为、有所后为，深入实施“五化”工程，推进55个城建项目，推动净畅绿亮美向更高层次迈进。彻底歼灭脏乱差，让城市更净。大力开展春季攻坚月、夏季提升月等活动，实现精细化、无死角道路保洁清扫模式。基本消除市区黑臭水体，启动“六河”综合治理和儿童公园提升改造工程。加快建设生活垃圾焚烧发电等5个垃圾处理项目。完成城区供水工程管线和净水厂主体施工，实施3处污水下穿铁路顶管工程。进一步完善路网，让城市更畅。推进浑江大街和长白山大街东延、北安大街和向阳桥改造以及5座公铁立交桥建设，打通向阳路等5条断头路，改造人行步道5万平方米。启动大规模增绿行动，让城市更绿。完成滨江景观带建设，新增绿地5万平方米，打造6个精品游园和1条特色街路，推进“300米见绿，500米见园”。实施点亮夜景工程，让城市更亮。推进“一江两岸”、临街风貌建筑、高层建筑和主要公建楼体亮化，完成市区主要桥梁和街路路灯升级改造。全方位提升城市品位，让城市更美。完成160栋老旧弃管小区综合治理和20栋临街楼体风貌改造，强化房地产市场管理，依法解决烂尾楼问题。实施江心喷泉等景观提升工程。改造各类棚户区8638户62.2万平方米。创新城市管理模式，打造数字化城管平台。

　　抓好县城和城镇建设。加快推进抚松镇城市出入口及道路景观建设、靖宇刘海山休闲广场、长白城市风貌改造、临江老旧小区污水管网改造等工程，启动抚松镇松城苑棚改，完成靖宇青龙河北岸道路及景观带建设、长白县城供水工程和中心街路改造，创建泉阳、长白等8个省级特色示范城镇。

　　抓好大交通体系建设。确保辉白高速白山段竣工通车，白临高速启动征地拆迁及部分路基工程，松长高速完成前期工作，力争沈白高铁白山段开工建设，白山机场纳入国家民航“十三五”开工建设规划调整范围并完成预可研，做好长白通用机场前期工作。推进错草至三道沟、靖宇至江源、漫江至桦树、松江河至泉阳公路建设，高标准做好白山至三道沟公路沿线绿化。建设农村公路139公里。加密路网，积极谋划鸭绿江界河及边防、口岸、革命老区等专项公路建设。

　　（五）加大生态环境保护力度，全力推进生态文明建设。牢固树立“绿水青山就是金山银山，冰天雪地也是金山银山”绿色发展理念，加快形成节约资源和保护环境的空间格局、产业结构、生产方式、生活方式，还自然以宁静、和谐、美丽。

　　加快推进绿色发展。构建市场导向的绿色技术创新体系，发展绿色金融，壮大节能环保产业、清洁生产产业、清洁能源产业。推进能源生产和消费革命，构建清洁低碳、安全高效的能源体系。促进资源全面节约和循环利用，降低能耗、物耗，实现生产系统和生活系统循环链接。倡导简约适度、绿色低碳的生活方式，反对奢侈浪费和不合理消费，开展创建节约型机关、绿色家庭、绿色学校、绿色社区和绿色出行等行动。

　　强化生态系统保护。推进长白山森林生态系统修复工程，全面完成林地清收还林任务，人工造林6000亩，森林抚育79.6万亩。谋划浑江、鸭绿江、头道松花江等740个“三江源”山水林田湖生态保护修复项目，推进砟子、八宝等一批矿山治理项目，构建浑江生态廊道。大力推进生态创建工作，打造6个省级生态乡镇、12个生态村、4个绿美示范村屯。抓好中央环保督察反馈意见整改，开展自然保护区、矿区、饮用水水源保护区生态环境综合整治“回头看”。推进环保系统监测监察执法垂直管理改革，加强环境执法，严厉打击环境违法犯罪行为。

　　加大污染防治力度。坚持全民共治、源头防治，集中力量推进大气、水、土壤污染防治攻坚战。打好蓝天保卫战，开展工业粉尘、城市扬尘、机动车尾气专项整治和燃煤锅炉改造，市区空气优良天数力争达到310天。深入推行“河长制”，加大重点流域重要支流水环境治理力度，确保“三江”干流水质优良比例70%以上。强化土壤污染管控和修复，加强农业面源污染防治，开展农村人居环境整治行动。加大固体废弃物和垃圾处置力度。严格污染排放标准，强化排污者责任，健全环保信用评价、信息强制性披露、严惩重罚等制度。

　　（六）激发文化创新创造活力，推动文化繁荣发展。坚持中国特色社会主义文化发展道路和“二为”方向、“双百”方针、“双创”原则，深度挖掘白山文化底蕴，激发文化创新创造活力，提升文化软实力。

　　大力开展社会主义核心价值观教育。实施社会主义核心价值观宣传普及行动，弘扬民族精神和时代精神，加强爱国主义、集体主义、社会主义教育，构建以社会主义核心价值观为引领的大中小幼一体化德育体系，将社会主义核心价值观融入社会各领域，转化为人们的情感认同和行为习惯。积极推动文艺创新，重点围绕重大革命历史题材和现实题材，推出更多具有原创价值和自主知识产权的广播电视文艺精品力作。加强社会公德、职业道德、家庭美德、个人品德建设，持续开展“白山好人”评选，有效提升全社会文明程度。

　　打造文化事业和文化产业发展新高地。深化文化体制改革，构建经济社会效益相统一的体制机制。加快建设文化体育艺术中心、靖宇东北抗联教育基地，组织开展以贯彻落实党的十九大精神、庆祝改革开放40周年为主题的系列文化活动。深入实施文化惠民工程，开展送文化下乡，丰富群众性文化活动。加强文物保护利用和文化遗产保护传承。广泛开展全民健身活动，完善公共体育设施，积极参加省第十八届传统体育运动会，筹办好省第八届少数民族运动会，全力申办国际少年冬运会。积极培育文化市场主体，大力发展新兴文化业态，推进鑫德文化街等一批文化产业项目。

　　（七）全面加强和创新社会治理，加快平安白山建设。加强社会治理制度和体制建设，不断提高社会治理社会化、法治化、智能化、专业化水平。

　　扎实推进安全发展。弘扬生命至上、安全第一的思想，完善安全生产责任制，坚决遏制重特大安全事故。始终保持高压严管态势，强化煤矿分类监管，巩固成果、细化措施，推进安全生产标准化建设，提升安全基础条件和事故防范能力。加大非煤矿山、道路交通、建筑施工、危化品、消防等重点领域整治力度。深入推进安全生产领域改革发展和“五个一工程”，实现五大行业重点企业全覆盖。持续推进食品药品安全放心工程，严守从农田到餐桌每一道防线，保障群众“舌尖上的安全”。提升防灾减灾救灾和应急处置能力，实现连续39年无重大森林火灾。

　　强化社会治理。大力推进“七五”普法，推行法律顾问制度，加强法律援助，扩大“两公”律师覆盖面。加快建设立体化、信息化社会治安防控体系，依法严厉打击黄赌毒黑拐骗等违法犯罪活动，全面深化“三打击一整治”专项行动，保持命案现案破案率100%，保护人民人身权、财产权、人格权。加强互联网安全管理，强化舆情应对处置，营造清朗的网络空间。开展“人民调解防护网”工程、“百姓说事点”建设和民间纠纷“零激化”行动，构建多元化矛盾化解体系。开展“重点信访案件攻坚年”活动，推进法治信访、阳光信访和责任信访，及时就地解决群众合理合法信访诉求。强化“三道防线”，完善“五位一体”、“六边管控”机制，确保边境安全稳定。

　　（八）坚持以人民为中心发展思想，努力提高保障改善民生水平。抓住人民最关心最直接最现实的利益问题，既尽力而为，又量力而行，一件事情接着一件事情办，一年接着一年干。坚持人人尽责、人人享有，不断满足人民日益增长的美好生活需要，使人民获得感、幸福感、安全感更加充实、更有保障、更可持续。

　　扎实推进精准扶贫、精准脱贫。把脱贫攻坚作为重大政治任务和最大民生工程，以产业扶贫为根本，以健康扶贫为重点，注重扶贫、扶志、扶智相结合，深入实施十大工程，落细落准落实攻坚任务，推进130个特色种养殖、光伏等产业扶贫项目，60个村屯硬化路、农田水利等基础设施扶贫项目，集中攻破水、路、电、房等难点。全面推行农村贫困人口县域内先诊疗后付费“一站式”结算制度和“帮扶明白卡”上墙，推进扶贫政策“落地入户”专项行动，有效提升“三率一度”，确保脱真贫、真脱贫。脱贫7000人以上，退出53个贫困村，长白县脱贫摘帽。

　　加强社会保障体系建设。按照兜底线、织密网、建机制要求，加快建设覆盖全民、城乡统筹、权责清晰、保障适度、可持续的多层次社会保障体系。全面实施全民参保计划，实现社保“一卡通”全覆盖。大力实施“创业引领”、“就业扶持”工程，统筹做好高校毕业生、去产能职工等重点群体就业工作，城镇新增就业3.8万人，农村劳动力转移就业10万人，确保零就业家庭动态“清零”。加大困境儿童救助关爱力度。构建居家为基础、社区为依托、机构为补充、医养结合的养老服务体系。

　　全面发展社会事业。办好9方面34件民生实事。优先发展教育事业，进一步优化布局、提升质量，新增3所公办幼儿园，开工建设长白山国际旅游职业学院，长白山职业技术学院新校区竣工投入使用，职业技能培训公共实训基地实现国家批复的年实训能力。深入实施健康白山战略，完善家庭医生签约服务制度，抓好分级诊疗和远程会诊建设，建成市中心医院全科医生培训基地，县域内就诊率达到90%以上，基本实现大病不出县。推进兴边富民行动，推动民族地区和民族事业繁荣发展。依法加强宗教事务管理，抓好老龄、妇女儿童、残疾人、人防、外事侨务、档案、地方志等工作。

　　三、以习近平新时代中国特色社会主义思想为统领，全面加强政府自身建设

　　中华民族伟大复兴，绝不是轻轻松松、敲锣打鼓就能实现的。白山绿色转型全面振兴，同样需要我们付出更为艰巨、更为艰苦的努力和奋斗。为此，我们必须加强政府自身建设，打造一支忠诚干净担当的政府系统干部队伍。

　　（一）坚定不移增强“四个意识”，坚决维护党的核心和党中央权威。把学习十九大精神作为当前首要的政治任务，按照学懂弄通做实的要求，进一步掀起学习贯彻十九大精神热潮。旗帜鲜明讲政治，切实增强“四个意识”，坚决拥戴党的核心，坚持维护党的核心，坚定执行党的政治路线，在政治立场、政治方向、政治原则、政治道路上同以习近平同志为核心的党中央保持高度一致，不断提高政治觉悟和政治能力。坚定不移守规矩，坚决贯彻民主集中制，认真执行重大事项请示报告制度，不断增强党内政治生活的政治性、时代性、原则性、战斗性。永葆忠诚敢担当，围绕推进“五大建设”、实施“七大战略”、打好“三大攻坚战”，以功成不必在我的境界和胸怀，多做打基础、利长远的事，努力创造经得起历史和人民检验的业绩，用政府工作成效体现忠诚、展现担当。

　　（二）坚持科学依法民主决策，努力打造人民满意的服务型政府。坚持科学决策，深入一线调研，完善政府决策和议事规则，健全重大决策听证、咨询和公示制度。坚持依法决策，进一步落实重大执法决定法制审核和“双随机一公开”，严格规范公正文明执法，积极配合做好城乡建设管理、环境和历史文化保护等方面立法工作。坚持民主决策，自觉接受人大、政协和司法、社会各界监督，主动听取民主党派、工商联、无党派人士和人民团体意见建议，认真办理人大议案、建议和政协提案。加大政务公开力度，保障人民群众知情权、参与权、表达权和监督权。优化公共服务，持续改进作风，察民情、体民意、解民忧，千方百计提升公共服务质量和效率。实施“软环境建设提升年”，激发和保护企业家精神，大力营造重商、安商、亲商、富商氛围，人人成为软环境的建设者、服务者、贡献者。

　　（三）加强学习和实践，努力在增强“八个本领”上下功夫。始终把政治过硬、本领高强作为加强政府系统队伍建设的重要遵循和根本要求。增强学习本领，缺什么学什么，主动向先进理念学、向先进地区学、向先进做法学、向身边典型学、向实践学，建设学习型机关、学习型队伍；增强政治领导本领，坚持战略思维、创新思维、辩证思维、法治思维、底线思维，坚决执行党的路线方针政策，把省委、省政府和市委各项决策部署落到实处；增强改革创新本领，保持锐意进取的精神风貌，善于结合实际创造性推动工作，善于运用互联网技术和信息化手段开展工作；增强科学发展本领，善于贯彻新发展理念，不断开创发展新局面；增强依法执政本领，用法治思维谋划发展、破解难题；增强群众工作本领，创新群众工作体制机制和方式方法；增强狠抓落实本领，坚持说实话、谋实事、出实招、求实效，把雷厉风行和久久为功有机结合起来，勇于攻坚克难，以钉钉子精神做实做细做好各项工作，特别是针对表态多调门高、行动少落实差等形式主义、官僚主义突出问题，拿出过硬措施，扎扎实实地改；增强驾驭风险本领，建立健全各方面风险防控机制，善于处理各种复杂矛盾，勇于战胜前进道路上的各种艰难险阻，牢牢把握工作主动权。

　　（四）全面贯彻新时代党的建设总要求，将全面从严治党和反腐败“一岗双责”任务落到实处。按照新时代党的建设总要求，认真履行全面从严治党责任，深入贯彻中央八项规定实施细则精神，抓好政府系统党风廉洁建设。始终把党的政治建设摆在首位，思想建党和制度治党同向发力，统筹推进政府系统党的各项建设，抓住“关键少数”，扎实推进“两学一做”学习教育常态化制度化，积极投身“不忘初心、牢记使命”主题教育。持之以恒正风肃纪，坚持以上率下，持续整治“四风”，巩固和拓展作风建设成果。坚决查处侵害群众利益的不正之风和腐败问题，加强对扶贫、工程招投标、行政审批、政府采购等重点领域监管，推进廉洁文化建设，强化不敢腐的震慑，扎牢不能腐的笼子，增强不想腐的自觉，切实做到为民务实清廉。

　　各位代表！新时代，新使命，新气象。党的十九大为我们描绘了“两个一百年”和中华民族伟大复兴的宏伟蓝图，白山绿色转型全面振兴展示了勃勃生机，进入了崭新阶段。让我们更加紧密地团结在以习近平同志为核心的党中央周围，在省委、省政府和市委的正确领导下，不忘初心、牢记使命，以更高的境界、更强的本领、更优的作风、更好的精神状态，不断开创白山绿色转型全面振兴新局面，以优异成绩向改革开放40周年献礼，为实现“两个一百年”奋斗目标和中华民族伟大复兴的中国梦贡献白山力量！
